# Epidemiological Study on the Interaction between the *PNPLA3* (rs738409) and Gut Microbiota in Metabolic Dysfunction-Associated Steatotic Liver Disease

**DOI:** 10.3390/genes15091172

**Published:** 2024-09-06

**Authors:** Satoshi Sato, Chikara Iino, Takafumi Sasada, Go Soma, Keisuke Furusawa, Kenta Yoshida, Kaori Sawada, Tatsuya Mikami, Shigeyuki Nakaji, Hirotake Sakuraba, Shinsaku Fukuda

**Affiliations:** 1 Department of Gastroenterology, Hematology, and Clinical Immunology, Hirosaki University Graduate School of Medicine, Hirosaki 036-8562, Japan; 2 Department of Preemptive Medicine, Hirosaki University Graduate School of Medicine, Hirosaki 036-8562, Japan

**Keywords:** MASLD, SNPs, PNPLA3, gut microbiota

## Abstract

Many factors are associated with the development and progression of metabolic dysfunction-associated steatotic liver disease (MASLD); however, genetics and gut microbiota are representative factors. Recent studies have highlighted the link between host genes and the gut microbiota. Although there have been many studies on the separate effects of single nucleotide polymorphisms (SNPs) and gut bacteria on MASLD, few epidemiological studies have examined how SNPs and gut bacteria interact in the development and progression of MASLD. This study aimed to investigate the association between *PNPLA3* rs738409, a representative MASLD-related SNP, and gut bacteria in MASLD using a cross-sectional study of the general population. The 526 participants (318 normal and 208 MASLD groups) were grouped into the *PNPLA3* rs738409 SNP, CC, CG, and GG genotypes, and the differences in the gut microbiota were investigated in each group. The *PNPLA3* rs738409 CC and CG genotypes were associated with decreased *Blautia* and *Ruminococcaceae* in the MASLD group. They were negatively correlated with controlled attenuation parameter levels, body mass index, serum blood glucose, and triglycerides. In contrast, there was no association between the normal and MASLD groups and the gut bacteria in the *PNPLA3* rs738409, the GG genotype group. This finding implies that dietary interventions and probiotics may be more effective in preventing and treating MASLD in individuals with the *PNPLA3* rs738409 CC and CG genotypes. In contrast, their efficacy may be limited in those with the GG genotype.

## 1. Introduction

Metabolic dysfunction-associated steatotic liver disease (MASLD) is a hepatic phenotype of metabolic syndrome with an increasing prevalence of approximately 30% and increasing [1]. When MASLD was renamed non-alcoholic fatty liver disease (NAFLD) in 2023, the diagnostic criteria included at least one cardiometabolic criteria in addition to fatty liver [2]. Although various factors, such as sex, age, body size, diet, and lifestyle, are intricately related to the onset and progression of MASLD, the significant factors are genes and gut microbiota [3,4,5]. The prevalence of MASLD in Japan is 29.7%, which is lower than that in Europe and the U.S. However, the prevalence of lean MASLD is considered high in the Asian region, including Japan [6,7,8].

Recently, genome-wide association studies (GWASs) have identified many single nucleotide polymorphisms (SNPs) in NAFLD susceptibility genes. In 2008, Romeo reported the patatin-like phospholipase domain-containing 3 (*PNPLA3*) gene [9]. *PNPLA3* rs738409 (C > G) has been associated with NAFLD in many ethnic groups, including the Japanese [10,11,12,13,14]. In addition to *PNPLA3*, various SNPs have been reported to be associated with NAFLD [15,16]. Among the many NAFLD-related SNPs, the *PNPLA3* rs738409 SNP (C > G) is common in Japan, and the overall Japanese prevalence of the GG genotype is reported to be approximately 20% and 40% in NAFLD patients [11,16,17].

The gut microbiota is closely involved in developing liver fat and fibrosis, and the gut microbiota and liver association is called the gut–liver axis [18,19]. Several studies have investigated the relationship between gut bacteria and NAFLD [20,21,22].

Recent studies have highlighted a link between host genes and gut microbiota [23,24,25,26] Cystic fibrosis patients have a 5–10-fold increased risk of colorectal cancer, but *Actinobacteria* and *Clostridium*, which are increased in cystic fibrosis, are also known to be increased in colorectal cancer patients [27]. It has been suggested that a predisposition to developing colorectal cancer in cystic fibrosis patients may be associated with an increased abundance of *Actinobacteria* and *Clostridium*, accompanied by downregulation of the host’s *CFTR* and *HPGD* genes. A common set of host genes and pathways involved in gastrointestinal inflammation, gut barrier protection, and energy metabolism have also been reported to be associated with disease-specific gut bacteria [25]. Although studies examining this link are limited and have primarily focused on Western populations, recent research on Japanese individuals has revealed that host genetic factors, including SNPs, can significantly impact the gut microbiota composition [28,29].

Although there have been many previous studies on the separate effects of SNPs and gut bacteria on MASLD, few epidemiological studies have examined how SNPs and gut bacteria interact during the development and progression of MASLD. In this study, we investigated the association between *PNPLA3* rs738409, a representative MASLD-related SNP, and gut bacteria in MASLD using a cross-sectional study of the general population.

## 2. Materials and Methods

### 2.1. Study Participants

**Figure 1:**
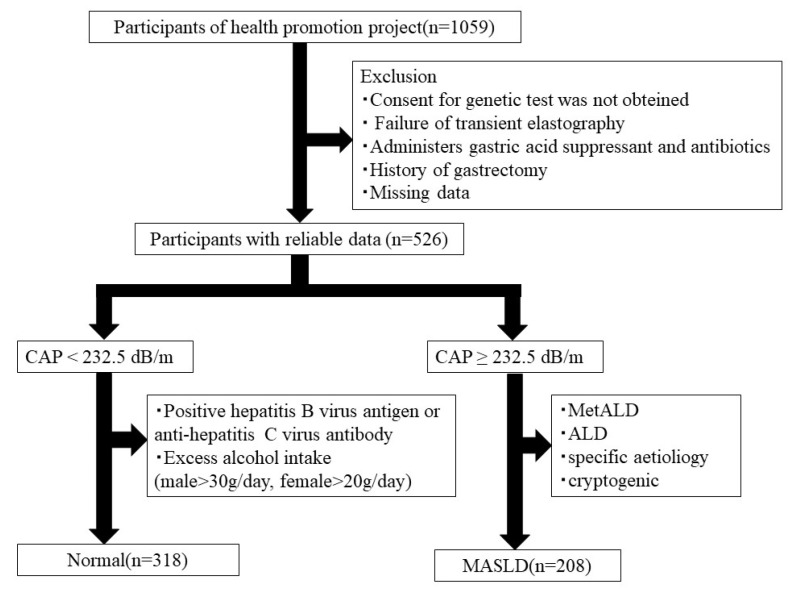
Study enrollment flowchart. CAP, controlled attenuation parameter; MASLD, metabolic dysfunction-associated steatotic liver disease; and ALD, alcohol-associated liver disease.

This study was part of the Iwaki Health Promotion Project, a community-based health promotion project for the general Japanese population. It is conducted annually in June as a regular health checkup for residents of the Iwaki area of Hirosaki City, Aomori Prefecture [30]. All the participants voluntarily responded to a public call for participation. A total of 1059 adults (aged 19–88 years) participated in the study. Participants who could not give consent for genetic testing, those who could not diagnose steatotic liver disease (SLD) due to failure of transient elastography measurement, and those who had one or more missing values in any of the measures were excluded. Additionally, those who had undergone gastrectomy or were taking gastric suppressant were excluded, as oral and gut microbiota differ significantly due to gastric acid sterilization, and this relationship may change significantly if gastric acid secretion is reduced. Furthermore, we excluded participants who were taking antibiotics because antibiotic use can drastically change the composition of the gut microbiota. Based on previous reports, SLD was diagnosed with a cutoff value of 232.5 dB/m, the CAP value of FibroScan (Echosens, Paris, France) [31]. Since hepatitis B and C and alcohol consumption are known to significantly affect the intestinal environment, we defined a normal group of 318 participants by excluding individuals with HBs antigen positivity, anti-HCV antibody positivity, and excessive alcohol consumption (≥30 g/day for males, ≥20 g/day for females) from the non-SLD group [32,33,34]. In the SLD group, 208 patients who met the diagnostic criteria were included in the MASLD group (Figure 1). In total, 526 patients (318 in the normal group and 208 in the MASLD group) were included in the analysis.

### 2.2. Transient Elastography

The controlled attenuation parameter (CAP) and liver stiffness measurement (LSM) were performed using a Fibroscan530 (Echosens, Paris, France) with the M and XL probes. All examinations were performed by five hepatologists who underwent specialized training. When the number of measurements was < 10, or the ratio of the interquartile range was >0.30, the measured values were excluded due to unreliability. Previous studies defined steatosis as a CAP value >232.5 dB/m [31].

### 2.3. Clinical Parameters

The following clinical parameters were recorded on the same day as the transient examination: sex, age, height, body mass index (BMI; calculated by dividing the weight in kg by the squared height in m), waist circumference, results of HBs antigen or anti-HCV tests, and levels of aspartate aminotransferase (AST), alanine aminotransferase (ALT), γ-glutamyl transpeptidase, glucose, hemoglobin A1c (HbA1c), high-density lipoprotein (HDL) cholesterol, low-density lipoprotein (LDL) cholesterol, and triglycerides.

The FIB-4 index was calculated as follows:

{age × AST (U/L)}/{blood platelet count (109/L) × √ALT (U/L)]}.

The aspartate aminotransferase to platelet ratio (APRI) was calculated as follows.

{[AST/ULN]/platelet count (× 109/L)} × 100.

The NAFLD fibrosis (NFS) score was calculated as follows.

−1.675 + 0.037 × age (years) + 0.094 × BMI (kg/m2) + 1.13 × diabetes mellitus (yes = 1, no = 0) + 0.99 × AST

(U/l)/ALT (U/;) −0.013 × platelet counts (104/µL) −0.66 × albumin (g/dL).

The FibroScan-aspartate aminotransferase (FAST) score was calculated as follows [35]:

{exp (–1.65 + 1.07 × ln (LSM) + 2.66 × 10 − 8 × CAP3 − 63.3 × AST − 1)}/{1 + exp (–1.65 + 1.07 × ln (LSM) + 2.66 × 10 − 8 × CAP3 − 63.3 × AST − 1)}

### 2.4. MASLD Diagnosis

Participants with fatty liver who met any of the following cardiometabolic criteria were diagnosed with MASLD: obesity/central obesity, hyperglycemia or diabetes, high blood pressure, high triglyceride levels, and reduced HDL cholesterol were diagnosed with MASLD [2]. The specific criteria included a BMI ≥ 23 kg/m^2^ or waist circumference ≥ 94 cm for males and ≥80 cm for females; fasting blood glucose ≥ 100 mg/dL, postprandial blood glucose ≥ 140 mg/dL, HbA1c ≥ 5.7%, or undergoing treatment for type 2 diabetes; blood pressure ≥ 130/85 mmHg or currently undergoing antihypertensive treatment; triglycerides ≥ 150 mg/dL or currently undergoing treatment for dyslipidemia; and HDL cholesterol ≤ 40 mg/dL for males and ≤50 mg/dL for females.

### 2.5. DNA Preparation and SNP Genotyping

SNP genotypes were determined by whole-genome sequencing with imputation from the Japonica Array (Toshiba, Tokyo, Japan), which consists of population-specific SNP markers designed from the 1070 whole-genome reference panels and TaqMan PCR [36,37]. Whole genome sequencing and imputation were performed by Takara Bio Corporation (Shiga, Japan) and Toshiba Corporation, respectively. For the Japonica Array, DNA was purified from peripheral whole blood using a QIAamp.^R^96 DNA Blood Kit (QIAGEN, Hilden, Germany) and extracted from plasma pellets for whole-genome sequencing. Among the many SNPs extracted by the Japonica Array, this study focused on SNP *PNPLA3* rs738409, which has been reported to be most involved in the onset and progression of MASLD in previous studies [10,11,12,14].

### 2.6. Measurements of the Gut Microbiota

Gut microbiota data were obtained following procedures. Fecal sample kits were distributed to the participants in advance, and fecal samples were collected at home. DNA was extracted from bead-beaten fecal suspensions using an automated nucleic acid extraction system (Precision System Science, Chiba, Japan). The MagDEA DNA 200 (GC) reagent kit (Precision System Science) was used for automated nucleic acid extraction. DNA extraction for all samples was completed within four months. Universal primer sets were used to amplify the V3–V4 region of the 16S rRNA gene. Solution preparation and condition setting for PCR amplification were performed according to the previous study [38]. PCR fragments purified using PCR Cleanup Filter Plates (Merck Millipore, Burlington, MA, USA) were quantified by real-time quantitative PCR (qPCR). To read DNA sequences, purified PCR fragments were analyzed by 2-cycle × 300-cycle paired-end sequencing on a MiSeq™ system (Illumina, San Diego, CA, USA). Paired-end reads were processed as follows: adapter sequences and low-quality bases (Q < 20) at the 3′ end of the reads were trimmed using Cutadapt (version: 1.13). Reads containing ambiguous bases N or shorter than 150 bases were excluded. Paired-end reads that met the criteria were merged into a single read called a “merged read”. Merged reads shorter than 370 base pairs or longer than 470 base pairs were excluded using the fastq_mergepairs subcommand of VSEARCH (version: 2.4.3) [39]. Furthermore, merged reads containing one or more identified sequencing errors were excluded. After removing chimeric reads detected by the uchime_denovo subcommand of VSEARCH, the remaining merged reads were clustered at a minimum sequence similarity of 97% to obtain operational taxonomic units (OTUs). Phylogenetic assignment of OTUs was performed by applying the RDP classifier (commit hash: 701e229dde7cbe53d4261301e23459d91615999d) based on their representative reads [40]. Predictions with a confidence score below 0.8 were treated as unclassified. The relative abundance of each bacterial genus in the gut microbiota was calculated by dividing the read count of each bacterial genus by the total read count.

### 2.7. Statistical Analysis

Categorical variables are presented as frequencies and continuous variables as medians, along with interquartile ranges. To compare differences in MASLD-related items and gut microbiota in CC, CG, and GG genotypes of *PNPLA3* rs738409, the Kruskal–Wallis and chi-square tests were used to compare the three groups. The microbiota was compared using linear discriminant analysis effect size (LEfse) [41]. Regression analyses were then used to evaluate the correlation between the relative abundance of individual bacterial species found to be associated with LEfSe and MASLD-related items. Pearson’s correlation coefficient was used to investigate the correlation between MASDL-related parameters and gut microbiota. A multiple regression model with MASLD-related items and gut microbiota was used for predictive analysis. Independent variables included sex, age, smoking habits, exercise habits, and medication for hypertension, dyslipidemia, or diabetes mellitus. Before simple correlation and multiple regression analyses, all continuous parameters were log-transformed (natural logarithm) to approximate a normal distribution.

Statistical analyses were performed using R software (R Foundation for Statistical Computing, version R-4.1.1) and the Statistical Package for the Social Sciences (SPSS) version 28.0 (SPSS Inc., Chicago, IL, USA). Statistical significance was set at *p* < 0.05.

### 2.8. Ethics Statement

This study was conducted per the ethical standards of the Declaration of Helsinki and approved by the Ethics Committee of Hirosaki University School of Medicine (approval number and date: 2018-012, approved on 11 May 2018). Informed consent was obtained from all participants.

## 3. Results

### 3.1. Participant Characteristics

Participants’ characteristics are presented in Table 1. The frequency of the *PNPLA3* rs738409 SNP in this study subjects was 28.5% for CC genotype, 49.4% for CG genotype, and 22.1% for GG genotype. The genotype frequencies calculated under the assumption of Hardy–Weinberg equilibrium were 28.3% for CC genotype, 49.8% for CG genotype, and 21.9% for GG genotype, which were almost the same as the actual values, indicating that Hardy–Weinberg equilibrium was established. The GG genotype of *PNPLA3* rs738408 was observed in 22.1% and 23.1% of all subjects and patients with MASLD, respectively. There was no significant association between *PNPLA3* rs738409 SNP and MASLD appearance in this study population. There were no significant differences in sex, age, smoking, or drinking habits between the three groups. Additionally, there were no differences in CAP or cardiometabolic criteria among the three groups. In contrast, AST and ALT levels were significantly higher in the GG genotype group than in the CC and CG genotype groups. Among the liver fibrosis scoring systems, the APRI and FIB-4 scores were higher in the GG genotype group than in the other groups.

Tables S1–S3 show the characteristics of the *PNPLA3* rs738409 SNP. The MASLD group was older than the control group for the CC and CG genotypes. There were significant differences in CAP values and cardiometabolic criteria between the normal and MASLD groups. However, there were no significant differences in sex, LSM, smoking, or exercise habits between the groups.

**Figure 2:**
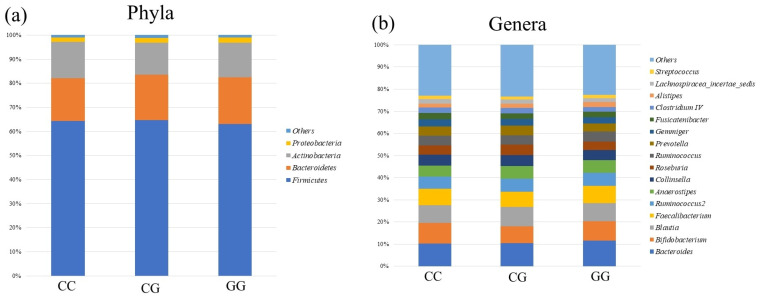
The relative abundances of gut microbiota in the *PNPLA3* rs738409 SNP groups at (**a**) the phyla and (**b**) genera level.

**Figure 3:**
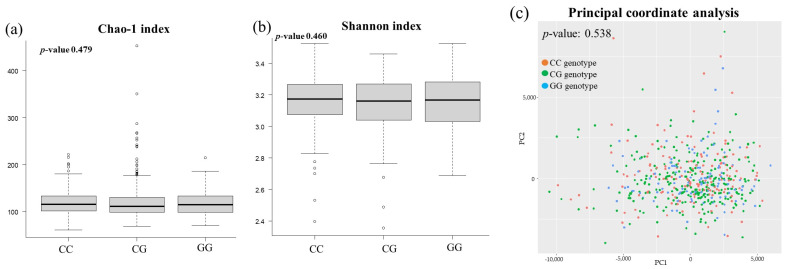
Comparison of the diversity of gut microbiota in the *PNPLA3* rs738409 SNP groups: (**a**) Chao-1 index, (**b**) Shannon index, (**c**) principal coordinate analysis.

Figure 2 and Figure 3 show the differences in the composition and diversity of the gut microbiota. There were no differences in composition between *PNPLA3* rs738409 SNP groups at either the phyla or genera levels (Figure 2). Figure 3 illustrates the diversity of the gut microbiome of the study subjects. Neither α diversity (as measured by the Chao-1 and Shannon indexes) nor β diversity (as assessed by principal coordinate analysis) showed significant differences across the *PNPLA3* rs738409 SNP groups.

### 3.2. Comparison of Gut Microbiota between Normal and MASLD Group

**Figure 4:**
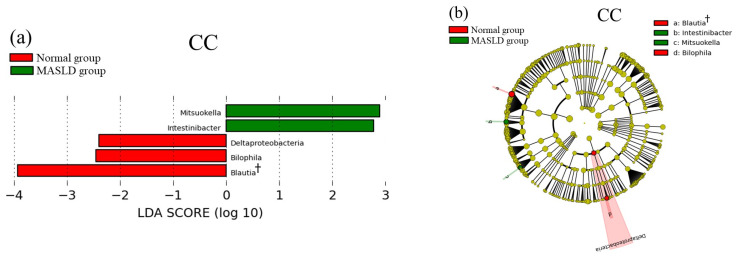

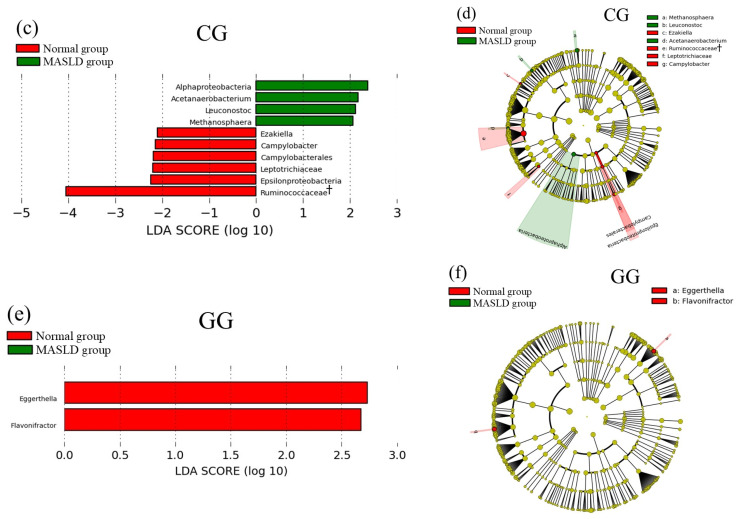
The LEfSe results of the oral microbiota in the *PNPLA3* rs738409 SNP groups (**a**) The linear discriminant in CC genotype. (**b**) The cladogram report in CC genotype. (**c**) The linear discriminant in CG genotype. (**d**) The cladogram report in CG genotype. (**e**) The linear discriminant in GG genotype. (**f**) The cladogram report in GG genotype. † relative abundance of 1% or more and an LDA score of 3 or more.

The LEfSe results for MASLD and gut microbiota are shown in Figure 4. Five bacterial taxa were significantly enriched in the CC genotype group, ten in the CG genotype, and two in the GG genotype. Among these, only two taxa had a relative abundance of 1% or more and an LDA score of 3 or more: *Blautia* in the CC genotype group (8.1% in the normal group and 5.6% in the MASLD group) and *Ruminococcaceae* in the CG genotype group (20.5% in the normal group and 16.9% in the MASLD group). The two taxa that showed differences in the GG group had extremely low relative abundances (<0.2%).

### 3.3. Comparison of Gut Microbiota among PNPLA3 rs738409 SNP

**Figure 5:**
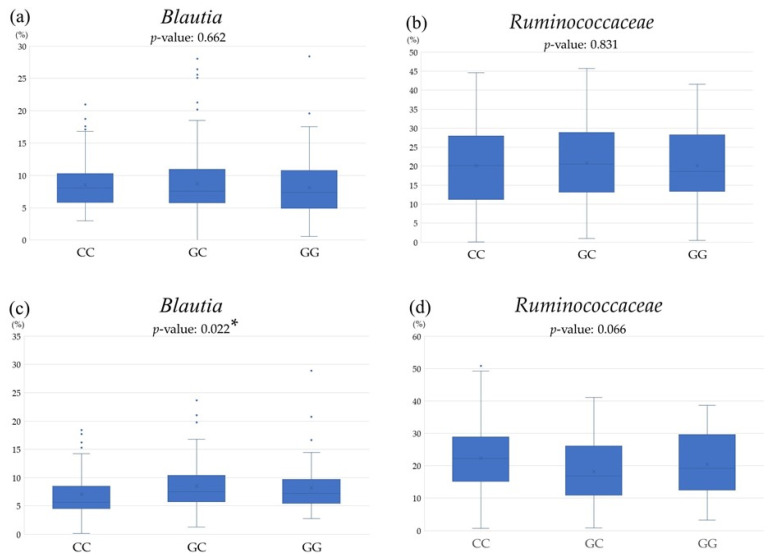
Comparison of gut microbiota among *PNPLA3* rs738409 SNP at (**a**) *Blautia* in normal group, (**b**) *Ruminococcaceae* in normal group, (**c**) *Blautia* in MASLD group, and (**d**) *Rumino coccaceae* in MASLD group. * *p*-value < 0.05.

Figure 5 presents the relative abundance of *Blautia* and *Ruminococcaceae* stratified by *PNPLA3* rs738409 genotypes, as determined by LEfSe analyses. There were no significant associations between either *Blautia* or *Ruminococcaceae* and the *PNPLA3* rs738409 SNP in the normal group. In contrast, the MASLD group showed a lower relative abundance of *Blautia* in the CC group, and a tendency for a decrease in *Ruminococcaceae* was noted in the CG group, although no statistically significant difference was observed.

### 3.4. Relationship between MASLD-Related Items and Gut Microbiota

Table 2 summarizes the correlation between MASLD-related items and gut microbiota in a single correlation analysis. In the CC genotype group, CAP and BMI had a negative correlation with the *Blautia*, and triglycerides had a negative correlation with *Ruminococcaceae*. In the CG genotype group, waist circumference and blood glucose had a negative correlation with *Blautia*, and CAP, waist circumference, and triglycerides had a negative correlation with *Ruminococcaceae*.

Next, multiple regression analysis was performed, where the dependent variables were MASLD-related items and the independent variables were sex, age, smoking and exercise habits, and medication of hypertension, dyslipidemia, or diabetes mellitus in addition to the gut microbiota. Table 3 presents the results of the study. The CC genotype group showed the same association as that of the single correlation group. In the CG genotype group, CAP and triglycerides were negatively correlated with *Ruminococcaceae*. In contrast, the GG group showed no significant correlation between MASLT-related items and gut microbiota in either univariate or multivariate correlations.

## 4. Discussion

This study found that the gut microbiota and MASLD groups differed according to the *PNPLA3* rs738409 SNPs. The gut microbiota’s impact on MASLD was more pronounced in individuals with the CC and CG genotypes than in those with the GG genotype. Our findings suggest that a decrease in acetate- and butyrate-producing bacteria, such as *Blautia* and *Ruminococcaceae*, may be involved in developing MASLD in *PNPLA3* rs738409 CC and CG genotypes.

The GG genotype of *PNPLA3* rs738409 was observed in 22.1% and 23.1% of all study participants and patients with MASLD, respectively. Previous studies have reported that approximately 20% of the Japanese population and 40% of patients with NAFLD carry the GG genotype of *PNPLA3* rs738409 [11,16,17]. While previous studies diagnosed NAFLD using liver biopsy, our study used FibroScan. Moreover, while a CAP value of 232.5 dB/m has been used as a cutoff for the diagnosis of SLD, some studies suggest a more stringent cutoff of 248 dB/m for SLD [31,42]. Differences in diagnostic methods and cutoff values may explain the varying prevalence of the *PNPLA3* GG genotype in our MASLD group compared with previous studies.

There was no significant association between *PNPLA3* rs738409 SNP and MASLD appearance in the study subjects. CAP values and cardiometabolic criteria included in the MASLD diagnostic criteria were also not associated with *PNPLA3* rs738409 SNP. In addition to genetic factors, many other factors contribute to the pathogenesis of MASLD, and the multi-parallel hit hypothesis proposes that organs other than the liver, such as the adipose tissue, oral cavity, and intestinal tract, as well as within the liver tissue, interactively contribute to the pathogenesis of MASLD [43]. In addition to the *PNPLA3* rs738409 SNP, many other SNPs have been associated with MASLD. In this study, no differences were observed in sex, age, body size, or lifestyle among the *PNPLA3* rs738409 genotypes. Still, it is possible that SNPs other than *PNPLA3* rs738409 or other factors, such as diet and lifestyle factors, may have contributed to the lack of association.

In contrast, ASL and ALT levels in this study were higher in the GG genotype group than in the CC and CG genotype groups. Furthermore, the APRI and FAST scores and liver fibrosis markers, including AST and ALT in their calculation formulas, were also higher in the GG genotype group. The *PNPLA3* rs738409 GG genotype increased AST and ALT levels by inducing hepatocyte inflammation [44,45]. It has been reported that inflammatory cytokines such as TNFα increase during the progression from simple steatosis to fibrosis in NAFLD [46,47]. *PNPLA3* rs738409 is strongly associated with liver injury, with the GG genotype reported to induce a necroinflammatory response approximately three times greater than that of the CC genotype [48]. Previous studies have suggested that the PNPLA3 rs738409 GG genotype may increase inflammatory cytokines such as TNFα and IL-6 in hepatocytes [46,49]. The *PNPLA3* rs738708 G type has been shown to enhance IL6/STAT3 activity in hepatocytes [50]. Additionally, *PNPLA3* has been reported to increase TNFα through NF-κB regulation [51]. Thus, *PNPLA3* is involved in triglyceride storage in hepatocytes and histological inflammation [48,52]. Our findings are consistent with those of previous studies and suggest that the *PNPLA3* rs738409 GG genotype group is more likely to experience liver damage than the CC or CG genotype groups.

In this study, the *PNPLA3* rs738409 CC and CG genotypes were associated with decreased numbers of *Blautia* and *Ruminococcaceae* in the MASLD group. They were negatively correlated with CAP level, BMI, serum blood glucose, and triglycerides. *Blautia* belongs to the *Lachnospiraceae* family and is more common in Japan than in other countries [53]. In animal studies, oral administration of *Blautia* has been shown to increase gut short-chain fatty acids and reduce high-fat obesity [54]. Epidemiological studies have reported that individuals with smaller visceral fat areas have increased levels of *Blautia* in their gut [55]. Animal studies have reported that *Blautia* increases the acetate concentration in the gut, inhibits hepatic steatosis and fibrosis, and suppresses the development of NAFLD/NASH [56]. Epidemiological studies have reported decreased levels of gut *Blautia* in NAFLD and MAFLD patients [57,58,59]. Furthermore, in this study, *Blautia* was negatively correlated with BMI in genotype CC and serum glucose in genotype CG, in addition to CAP levels, which supports previous studies showing that *Blautia* administration improves obesity and diabetes [60].

*Ruminococcaceae* is a superfamily of *Feacalibacterium*, *Gemmiger*, and *Ruminococcus* that produces butyric acid, a short-chain fatty acid that increases with dietary fiber intake [61,62]. *Ruminococcaceae* benefits the body by increasing intestinal butyrate levels, which are decreased in inflammatory bowel disease [63,64]. *Ruminococcaceae* are also associated with many liver diseases and are downregulated in NAFLD [57,58,65]. In this study, *Ruminococcaceae* showed a negative correlation with triglycerides in *PNPLA3* rs738409 CC and CG genotypes and with CAP values. Previous studies have also reported that *Ruminococcaceae* shows an inverse correlation with triglycerides [66]. In contrast, a study using Japanese gut bacteria and a Genome-Wide Association Study showed that *Ruminococcaceae* is affected by host genetic factors [29]. In this study, the MASLD group with *PNPLA3* genotypes CC and CG had decreased gut *Blatuia* and *Ruminococcaceae*, suggesting that decreased short-chain fatty acids, such as acetate and butyrate, may contribute to obesity, and increased blood glucose and triglyceride levels, which may contribute to the onset and progression of MASLD. In this study, there was no association between normal and MASLD gut bacteria in the *PNPLA3* rs738409 GG genotype. *PNPLA3* rs738409 is involved in the progression of MASLD by elevating inflammatory cytokines; however, gut bacteria are also associated with inflammatory cytokines. Gut *Blautia* and *Ruminococcaceae* negatively correlate with inflammatory cytokines such as TNAα and IL-6 [67,68].

In this study, individuals with the *PNPLA3* rs738409 GG genotype exhibited higher levels of AST and ALT as well as higher APRI and FAST scores, which incorporate AST and ALT into their calculation formulas, compared to those with the CC and CG genotypes. Although this study did not measure inflammatory cytokines, it is speculated that the increased levels of AST, ALT, APRI, and FAST in the *PNPLA3* rs738409 GG genotype group were due to hepatocyte damage caused by increased inflammatory cytokines. In this study, the *PNPLA3* rs738409 GG genotype group might have higher levels of pro-inflammatory cytokines than the CC and CG genotype groups, suggesting that the involvement of gut microbiota in MASLD may have been relatively small and not significantly different. In contrast, the CC and CG genotype groups of *PNPLA3* rs738409 showed relatively less liver injury caused by inflammatory cytokines than the GG genotype group, suggesting an association between gut bacteria and MASLD.

We found no significant differences in the relative abundance of *Blautia* and *Ruminococcaceae* between *PNPLA3* rs738409 SNP in the normal group. In contrast, the MASLD group showed decreased *Blautia* in the CC genotype and a trend towards decreased *Ruminococcaceae* in the CG genotype. In MASLD, dysbiosis of the intestinal tract is induced, which is called gut–liver axis [18,19]. It has been reported that both gut *Blautia* and *Ruminococcaceae* are decreased in MASLD patients [57,58,59,65]. The association between *PNPLA3* rs738409 and gut microbiota may be greater in MASLD patients in whom dysbiosis occurs than in the normal group.

In addition, LEfSe analysis revealed different bacterial taxa between the CC and CG genotypes of *PNPLA3* rs738409. However, both the CC and CG genotype groups were associated with MASLD-related items in *Blautia* and *Ruminococcaceae*. Although there was no significant difference between the CC and CG groups in terms of hepatic enzymes, it is possible that there was a subclinical level of inflammation between the CC group with no G-risk allele and the CG group with one G-risk allele that did not appear in the statistics. This may have resulted in the difference between the CC and CG groups.

In this study, *Blautia* and *Ruminococcaceae* were identified as gut bacteria associated with *PNPLA3* rs738409. A previous study on Japanese individuals investigating the relationship between host genetic factors and gut microbiota identified *Clostridiales*, *Ruminococcaceae*, *Erysipelotrichaceae*, *Lachnospiraceae*, *Feacalibacterium*, and *Ruminococcus* as being influenced by genetic factors [29]. On the other hand, there was no association between *Blautia* and host genetic factors, which was found to be associated in our study. Although both studies were conducted on Japanese populations, the previous study focused on healthy adults aged 20–64 living in various regions, while our study had a median age of 53 years living in the same area, resulting in somewhat different population characteristics for the two groups. Furthermore, although there are certain trends in the relationship between MASLD and gut microbiota, results vary significantly across studies [21,22,69]. Differences in age, eating habits, medications, ethnicity, MASLD diagnostic tools, and other factors may have contributed to the varied results across studies. While our findings generally support previous studies, some discrepancies were observed due to the presence of these confounding factors.

This study had several limitations. First, our study population was geographically limited to a district in Japan; therefore, our results cannot be generalized to all ethnicities. Second, gut microbiota is influenced by a variety of factors, including dietary habits and medication status, but this study did not fully adjust for these confounding factors, so caution is needed in interpreting the results. Third, fatty liver and liver fibrosis were diagnosed using FibroScan instead of a liver biopsy. An invasive liver biopsy, conducted as part of a general population health check, was not feasible in this study. Fourth, we did not measure the inflammatory cytokine levels. Although we speculated that inflammatory cytokines might have attenuated the association between MASLD and the gut microbiota in the *PNPLA3* GG genotype group, we did not measure these cytokines to support this hypothesis.

## 5. Conclusions

Our study suggests that individuals with the *PNPLA3* rs738409 CC and CG genotypes may be more susceptible to the influence of the gut microbiota on MASLD than those with the GG genotype. For individuals with the *PNPLA3* rs738409 CC or CG genotype, active consumption of dietary fiber and other rich foods that increase short-chain fatty acids may be beneficial for preventing and treating MASLD through an increase in gut *Blautia* and *Ruminococcaceae*. While dietary interventions may show less efficacy in individuals with the *PNPLA3* rs738409 GG genotype compared to those with the CC or CG genotypes, strict adherence to other lifestyle modifications, such as active exercise and no smoking, is crucial for preventing metabolic complications, including obesity, hypertension, diabetes, and dyslipidemia. Personalized medicine, such as prophylactic and therapeutic strategies based on the *PNPLA3* rs738409 SNP, is crucial.

## Figures and Tables

**Table 1:** Participant characteristics.

	CC	CG	GG	*p*-Value
	n =150	n = 260	n = 116	
MASLD	55 (36.7%)	105 (40.4%)	48 (41.4%)	0.684
Sex, male	43 (28.7%)	95 (36.5%)	40 (34.5%)	0.264
Age (year)	55.5 (43.8–66.0)	52.0 (39.0–65.8)	54.0 (39.0–66.0)	0.379
BMI (kg/m^2^)	22.1 (20.2–24.5)	22.8 (20.2–25.1)	22.0 (19.8–24.7)	0.326
Waist circumference (cm)	74.5 (67.5–81.0)	76.3 (69.3–84.6)	74.3 (69.0–83.3)	0.146
Fasting blood sugar (mmHg)	92.0 (86.0–98.3)	91.5 (86.0–99.0)	90.0 (84.3–97.0)	0.162
HbA1c (%)	5.7 (5.6–5.9)	5.7 (5.5–5.9)	5.6 (5.4–5.9)	0.269
Systolic blood pressure (mmHg)	122.0 (111.8–134.0)	122.5 (110.3–133.0)	122.5 (112.3–133.8)	0.750
Diastolic blood pressure (mmHg)	76.0 (69.0–82.3)	76.5 (69.0–84.0)	77.0 (69.0–85.0)	0.854
Triglycerides (mmHg)	76.5 (52.8–118.0)	76.5 (56.3–110.0)	80.5 (55.5–105.0)	0.960
HDL cholesterol (mmHg)	66.5 (53.0–76.0)	62.0 (51.3–77.0)	63.0 (54.3–75.8)	0.415
LDL cholesterol (mmHg)	121.5 (105.0–139.3)	117.0 (99.0–138.0)	114.0 (95.0–132.8)	0.105
Aspartate aminotransferase (IU/L)	21.0 (17.0–25.3)	20.0 (17.0–23.0)	22.0 (19.0–27.0)	<0.001 **
Alanine aminotransferase (IU/L)	17.0 (13.0–24.0)	17.0 (13.0–23.0)	20.0 (13.3–30.3)	0.031 *
γ-Glutamyl TransPeptidase (IU/L)	21.0 (15.0–28.5)	21.0 (15.0–33.5)	19.0 (15.0–30.8)	0.703
CAP (dB/m)	216.5 (179.0–260.0)	222.5 (190.3–260.8)	224.0 (178.0–267.8)	0.595
LSM (kPa)	4.3 (3.5–5.4)	4.2 (3.5–5.2)	4.4 (3.7–5.6)	0.787
Smoking habit	19 (12.7%)	40 (15.4%)	10 (8.6%)	0.196
Exercise habit	31 (20.7%)	36 (13.8%)	27 (23.3%)	0.050
Medication of hypertension	31 (20.7%)	50 (19.2%)	27 (23.3%)	0.668
Medication of diabetes mellitus	7 (4.7%)	12 (4.6%)	3 (2.6%)	0.623
Taking dyslipidemia	20 (13.3%)	22 (8.5%)	16 (13.8%)	0.177
Fatty liver index	13.4 (4.8–29.0)	14.1 (6.27–33.6)	9.8 (5.0–30.1)	0.344
APRI	0.19 (0.15–0.25)	0.19 (0.15–0.25)	0.23 (0.17–0.28)	0.005 **
FIB-4 index	0.95 (0.67–1.41)	0.91 (0.59–1.43)	1.00 (0.75–1.47)	0.276
FAST score	0.05 (0.03–0.10)	0.05 (0.03–0.09)	0.06 (0.04–0.14)	0.006 **
NFS	−2.05 (−2.97–1.11)	−2.0 (−3.14–0.90)	−2.09 (−3.15–1.04)	0.920

Number or median (range), MASLD, metabolic dysfunction associated with steatotic liver disease; BMI, body mass index; HbA1c, hemoglobin A1c; HDL, high-density lipoprotein; LDL, low-density lipoprotein; CAP, controlled attenuation parameter; LSM, liver stiffness measurement; APRI, aspartate aminotransferase to platelet ratio index; FAST score, FibroScan-aspartate aminotransferase score; NFS, nonalcoholic fatty liver disease fibrosis score. * *p*-value< 0.05, ** *p*-value < 0.01.

**Table 2:** The correlation between MASLD-related items and gut microbiota in the *PNPLA3* rs738409 SNP groups.

***Blautia***						
	**CC**		**CG**		**GG**	
	**r**	***p*-Value**	**r**	***p*-Value**	**r**	***p*-Value**
CAP	−0.181	0.026 *	−0.091	0.144	0.035	0.710
BMI	−0.179	0.028 *	−0.106	0.088	<0.001	0.998
Waist circumference	−0.146	0.076	−0.130	0.037 *	−0.048	0.606
Systolic blood pressure	−0.001	0.953	−0.018	0.770	−0.002	0.980
Diastolic blood pressure	0.047	0.570	0.013	0.840	−0.039	0.677
Blood glucose	−0.026	0.751	−0.176	0.005 **	0.129	0.169
HbA1c	−0.024	0.772	−0.106	0.090	−0.049	0.601
Triglycerides	−0.044	0.597	−0.064	0.307	−0.083	0.374
HDL cholesterol	0.128	0.118	−0.003	0.958	0.132	0.159
***Ruminococcaceae***						
	**CC**		**CG**		**GG**	
	**r**	***p*-Value**	**r**	***p*-Value**	**r**	***p*-Value**
CAP	0.069	0.401	−0.149	0.016 *	0.034	0.718
BMI	−0.029	0.728	−0.098	0.115	−0.084	0.372
Waist circumference	−0.057	0.490	−0.138	0.026 *	−0.026	0.783
Systolic blood pressure	−0.020	0.805	0.001	0.996	0.051	0.584
Diastolic blood pressure	−0.139	0.091	−0.092	0.140	−0.028	0.764
Blood glucose	−0.052	0.527	0.073	0.241	0.077	0.415
HbA1c	0.017	0.842	0.091	0.142	0.108	0.249
Triglycerides	−0.211	0.009 **	−0.217	<0.001 **	−0.162	0.082
HDL cholesterol	0.098	0.232	0.099	0.112	0.034	0.720

r, Pearson’s correlation coefficient; CAP, controlled attenuation parameter; BMI, body mass index; HbA1c, hemoglobin A1c; HDL, high-density lipoprotein. * *p*-value < 0.05, ** *p*-value < 0.01.

**Table 3:** Multiple analyses between MASLD-related items and gut microbiota in the *PNPLA3* rs738409 SNP groups.

***Blautia***									
	**CC**			**CG**			**GG**		
	**β**	***p*-Value**	**R^2^**	**β**	***p*-Value**	**R^2^**	**β**	***p*-Value**	**R^2^**
CAP	−0.195	0.031 *	0.050	−0.058	0.378	0.042	0.017	0.858	0.103
BMI	−0.198	0.028 *	0.051	−0.083	0.206	0.045	−0.001	0.992	0.103
Waist circumference	−0.188	0.058	0.043	−0.109	0.140	0.047	−0.076	0.505	0.106
Systolic blood pressure	0.015	0.865	0.018	0.052	0.455	0.041	−0.004	0.973	0.103
Diastolic blood pressure	0.055	0.526	0.021	0.056	0.391	0.041	−0.052	0.587	0.105
Blood glucose	−0.012	0.900	0.018	−0.149	0.061	0.052	0.139	0.184	0.051
HbA1c	−0.000	0.999	0.018	−0.074	0.360	0.042	−0.071	0.514	0.106
Triglycerides	−0.047	0.620	0.020	−0.039	0.571	0.040	−0.117	0.255	0.113
HDL cholesterol	0.151	0.105	0.036	−0.013	0.854	0.0.39	0.103	0.329	0.111
***Ruminococcaceae***									
	**CC**			**CG**			**GG**		
	**β**	***p*-Value**	**R^2^**	**β**	***p*-Value**	**R^2^**	**β**	***p*-Value**	**R^2^**
CAP	0.107	0.214	0.137	−0.170	0.008 **	0.109	0.017	0.857	0.124
BMI	0.010	0.905	0.128	−0.080	0.211	0.089	−0.000	0.999	0.124
Waist circumference	0.045	0.632	0.129	−0.107	0.138	0.091	0.111	0.322	0.132
Systolic blood pressure	−0.052	0.545	0.130	−0.079	0.242	0.088	0.143	0.169	0.139
Diastolic blood pressure	−0.139	0.090	0.145	−0.114	0.073	0.095	−0.012	0.902	0.124
Blood glucose	−0.047	0.602	0.129	0.030	0.700	0.084	0.110	0.287	0.133
HbA1c	−0.002	0.979	0.128	0.045	0.569	0.084	0.078	0.471	0.128
Triglycerides	−0.196	0.027 *	0.157	−0.217	<0.001 **	0.122	−0.180	0.074	0.150
HDL cholesterol	−0.040	0.646	0.129	−0.004	0.953	0.083	−0.051	0.626	0.126

The multivariate analysis was adjusted for age, sex, smoking habits, exercise habits, and medication for hypertension, dyslipidemia, or diabetes mellitus. β, standardized coefficient; R^2^, coefficient of determination; CAP, controlled attenuation parameter; BMI, body mass index; HbA1c, hemoglobin A1c HDL, high density lipoprotein. * *p*-value < 0.05, ** *p*-value < 0.01.

## Data Availability

The data presented in this study are available upon request from the corresponding author. The data are not publicly available due to privacy and ethical restrictions.

## Supplementary Materials

The following supporting information can be downloaded at: https://www.mdpi.com/article/10.3390/genes15091172/s1, Table S1 The characteristics in the CC genotype of PNPLA3 rs738409. Table S2 The characteristics in the CG genotype of PNPLA3 rs738409. Table S3 The characteristics in the GG genotype of PNPLA3 rs738409.
