# Supplementary material for: Epidemiological Study on the Interaction between the PNPLA3 (rs738409) and Gut Microbiota in Metabolic Dysfunction-Associated Steatotic Liver Disease
Source: Genes (Basel). 2024 Sep 6;15(9):1172. doi: 10.3390/genes15091172 (PMC11430940; doi:10.3390/genes15091172)
Supplement: Supplementary file 1 [file genes-15-01172-s001.zip › genes-3188347-supplementary.pdf]

Supplementary Table S1. Participant characteristics in the CC genotype of PNPLA3 rs738409.

| | Normal n=95 | MASLD n=55 | p-value |
|---|---|---|---|
| Sex, male | 24 (25.3%) | 19 (34.5%) | 0.306 |
| Age (year) | 51.0(42.0-64.0) | 60.0(45.0-68.0) | 0.033 |
| BMI ($kg/m^2$) | 21.2(19.3-22.7) | 24.2(22.5-26.1) | <0.001 |
| Waist circumference (cm) | 70.8(66.2-76.5) | 81.5(75.9-88.5) | <0.001 |
| Fasting blood sugar (mmHg) | 89.0(84.0-93.0) | 96.0(91.0-107.0) | <0.001 |
| HbA1c (%) | 5.6(5.5-5.8) | 5.9(5.7-6.2) | <0.001 |
| Systolic blood pressure (mmHg) | 119.0(110.0-130.0) | 125.0(116.0-137.0) | 0.030 |
| Diastolic blood pressure (mmHg) | 76.0(69.0-82.0) | 78.0(71.0-87.0) | 0.106 |
| Triglycerides (mmHg) | 66.0(50.0-97.0) | 96.0(69.0-148.0) | <0.001 |
| HDL cholesterol (mmHg) | 69.0(58.0-81.0) | 58.0(48.0-71.0) | <0.001 |
| LDL cholesterol (mmHg) | 119.0(100.0-133.0) | 132.0(111.0-149.0) | 0.007 |
| Aspartate aminotransferase (IU/L) | 21.0(17.0-25.0) | 21.0(17.0-27.0) | 0.578 |
| Alanine aminotransferase (IU/L) | 16.0(12.0-21.0) | 20.0(14.0-28.0) | <0.001 |
| γ-Glutamyl TransPeptidase (IU/L) | 18.0(14.0-26.0) | 21.0(19.0-33.0) | 0.012 |
| CAP (dB/m) | 194.0(158.0-215.0) | 271.0(252.0-312.0) | <0.001 |
| LSM (kPa) | 4.2(3.5-5.5) | 4.40(3.5-5.3) | 0.641 |
| Smoking habit | 8 (8.4%) | 11 (20.0%) | 0.072 |
| Exercise habit | 19 (20.0%) | 12 (21.8%) | 0.956 |
| Fatty liver index | 6.6(3.5-17.6) | 29.0(15.7-42.2) | <0.001 |
| APRI | 0.20(0.15-0.27) | 0.19(0.13-0.25) | 0.272 |
| FIB-4 index | 0.96(0.68-1.43) | 0.94(0.64-1.31) | 0.354 |
| FAST score | 0.05(0.02-0.08) | 0.08(0.04-0.13) | 0.003 |
| NFS | -2.20(-3.15—1.32) | -1.79(-2.84—0.91) | 0.153 |

Number or median (range), MASLD, metabolic dysfunction associated steatotic liver disease; BMI, body mass index; HDL, high density lipoprotein; LDL, low density lipoprotein; CAP, controlled attenuation parameter; LSM, liver stiffness measurement; APRI, aspartate aminotransferase to platelet ratio index; FAST score, FibroScan-aspartate aminotransferase score; NFS, non-alcoholic fatty liver disease fibrosis score.

Supplementary Table S2. Participant characteristics in the CG genotype of PNPLA3 rs738409.

| | Normal n=155 | MASLD n=105 | p-value |
|---|---|---|---|
| Sex, male | 50 (32.3%) | 45 (42.9%) | 0.107 |
| Age (year) | 50.0(37.0-64.0) | 57.0(44.0-66.0) | <0.001 |
| BMI (kg/m$^2$) | 21.0(19.2-23.4) | 24.6(22.9-26.9) | <0.001 |
| Waist circumference (cm) | 71.5(66.5-79.2) | 84.5(77.8-89.6) | <0.001 |
| Fasting blood sugar (mmHg) | 90.0(84.0-97.0) | 94.0(88.0-105.0) | <0.001 |
| HbA1c (%) | 5.6(5.4-5.8) | 5.7(5.6-6.1) | <0.001 |
| Systolic blood pressure (mmHg) | 117.0(108.0-129.0) | 127.0(114.5-139.0) | <0.001 |
| Diastolic blood pressure (mmHg) | 75.0(67.0-82.0) | 79.0(72.5-89.0) | <0.001 |
| Triglycerides (mmHg) | 66.0(49.0-95.0) | 97.0(65.5-136.0) | <0.001 |
| HDL cholesterol (mmHg) | 65.0(55.0-82.0) | 58.0(48.5-69.0) | <0.001 |
| LDL cholesterol (mmHg) | 111.0(94.0-129.0) | 126.0(110.0-143.5) | <0.001 |
| Aspartate aminotransferase (IU/L) | 19.0(17.0-23.0) | 21.0(18.0-24.5) | <0.001 |
| Alanine aminotransferase (IU/L) | 15.0(12.0-20.0) | 20.0(15.0-27.5) | <0.001 |
| γ-Glutamyl TransPeptidase (IU/L) | 19.0(15.0-29.0) | 25.0(16.5-38.5) | <0.001 |
| CAP (dB/m) | 195.0(-) | 278.0(250.0-303.0) | <0.001 |
| LSM (kPa) | 4.2(-) | 4.3(3.5-5.3) | 0.800 |
| Smoking habit | 21 (13.5%) | 19 (18.1%) | 0.411 |
| Exercise habit | 21 (13.5%) | 15 (14.3%) | 0.999 |
| Fatty liver index | 8.8(3.6-15.8) | 32.3(15.7-56.5) | <0.001 |
| APRI | 0.19(0.15-0.24) | 0.19(0.17-0.27) | 0.300 |
| FIB-4 index | 0.88(0.61-1.34) | 1.00(0.56-1.50) | 0.570 |
| FAST score | 0.04(0.02-0.07) | 0.07(0.04-0.12) | <0.001 |
| NFS | -2.32(-3.37--1.17) | -1.62(-2.62--0.57) | <0.001 |

Number or median (range), MASLD, metabolic dysfunction associated steatotic liver disease; BMI, body mass index; HDL, high density lipoprotein; LDL, low density lipoprotein; CAP, controlled attenuation parameter; LSM, liver stiffness measurement; APRI, aspartate aminotransferase to platelet ratio index; FAST score, FibroScan-aspartate aminotransferase score; NFS, non-alcoholic fatty liver disease fibrosis score.

Supplementary Table S3. Participant characteristics in the GG genotype of PNPLA3 rs738409.

| | Normal n=68 | MASLD n=48 | p-value |
|---|---|---|---|
| Sex, male | 22 (32.4%) | 18 (37.5%) | 0.707 |
| Age (year) | 52.5(38.0-64.0) | 57.0(44.3-68.0) | 0.148 |
| BMI (kg/m²) | 21.3(-) | 23.5(21.3-26.7) | <0.001 |
| Waist circumference (cm) | 71.6(66.6-78.5) | 80.7(72.0-87.4) | <0.001 |
| Fasting blood sugar (mmHg) | 87.0(83.0-92.0) | 94.5(87.5-102.8) | <0.001 |
| HbA1c (%) | 5.6(5.4-5.8) | 5.8(5.6-6.3) | <0.001 |
| Systolic blood pressure (mmHg) | 119.5(109.0-132.5) | 126.0(120.0-136.5) | 0.023 |
| Diastolic blood pressure (mmHg) | 77.0(68.3-84.0) | 78.0(70.0-85.0) | 0.309 |
| Triglycerides (mmHg) | 70.5(50.0-91.8) | 95.5(59.8-124.0) | 0.001 |
| HDL cholesterol (mmHg) | 65.5(56.0-76.0) | 61.0(48.3-74.8) | 0.071 |
| LDL cholesterol (mmHg) | 110.0(88.0-131.8) | 115.5(107.0-135.3) | 0.060 |
| Aspartate aminotransferase (IU/L) | 21.5(18.3-25.0) | 25.5(19.3-30.8) | 0.018 |
| Alanine aminotransferase (IU/L) | 17.5(13.0-23.0) | 23.0(17.0-44.0) | <0.001 |
| γ-Glutamyl TransPeptidase (IU/L) | 18.0(14.0-26.0) | 27.0(16.0-45.5) | 0.003 |
| CAP (dB/m) | 185.0(155.0-208.0) | 278.5(255.5-301.3) | <0.001 |
| LSM (kPa) | 4.2(3.7-5.0) | 4.5(3.7-6.1) | 0.136 |
| Smoking habit | 5 (7.4%) | 5 (10.4%) | 0.808 |
| Exercise habit | 15 (22.1%) | 12 (25.0%) | 0.884 |
| Fatty liver index | 6.7(4.1-15.6) | 22.4(9.4-49.8) | <0.001 |
| APRI | 0.23(0.17-0.27) | 0.24(0.18-0.31) | 0.061 |
| FIB-4 index | 0.98(0.72-1.53) | 1.02(0.78-1.47) | 0.523 |
| FAST score | 0.06(0.03-0.08) | 0.12(0.04-0.22) | <0.001 |
| NFS | -2.42(-3.45--1.63) | -1.86(-2.30--0.78) | 0.015 |

Number or median (range), MASLD, metabolic dysfunction associated steatotic liver disease; BMI, body mass index; HDL, high density lipoprotein; LDL, low density lipoprotein; CAP, controlled attenuation parameter; LSM, liver stiffness measurement; APRI, aspartate aminotransferase to platelet ratio index; FAST score, FibroScan-aspartate aminotransferase score; NFS, non-alcoholic fatty liver disease fibrosis score.
